# Accessibility of essential anticancer medicines for children in the Sichuan Province of China

**DOI:** 10.3389/fpubh.2022.980969

**Published:** 2022-11-04

**Authors:** Zhe Chen, Siyu Li, Kun Zou, Hailong Li, Linan Zeng, Xiaoxi Lu, Zhi-Jun Jia, Guo Cheng, Lingli Zhang

**Affiliations:** ^1^Department of Pharmacy, West China Second University Hospital, Sichuan University, Chengdu, China; ^2^Evidence-Based Pharmacy Center, West China Second University Hospital, Sichuan University, Chengdu, China; ^3^NMPA Key Laboratory for Technical Research on Drug Products In Vitro and In Vivo Correlation, Chengdu, China; ^4^Key Laboratory of Birth Defects and Related Diseases of Women and Children, Ministry of Education, Chengdu, China; ^5^West China School of Pharmacy, Sichuan University, Chengdu, China; ^6^West China School of Medicine, Sichuan University, Chengdu, China; ^7^Department of Pediatrics, West China Second University Hospital, Sichuan University, Chengdu, China; ^8^Laboratory of Molecular Translational Medicine, Center for Translational Medicine, Sichuan University, Chengdu, China

**Keywords:** child, anticancer medicine, essential medicine, accessibility, availability, affordability, price

## Abstract

**Background:**

Compared with high-income countries, the survival rate of childhood cancer is lower in low- and middle-income countries. Access to essential anticancer medicines is an indispensable component of pediatric cancer treatment, which is still a big challenge in low- and middle-income countries.

**Objective:**

To assess the accessibility of essential anticancer medicines for children in public hospitals in the Sichuan Province of China.

**Methods:**

Based on the data of the Sichuan Province Drug Use Monitoring Platform in 2020, a retrospective study was conducted to investigate the original brands and generics of 34 anticancer and three supportive essential medicines for children (a total of 97 specific strengths) in Sichuan Province. The availability, price, and affordability of surveyed medicines were evaluated in all 152 tertiary public hospitals (120 general hospitals, 31 children's hospitals, and one cancer hospital) that could diagnose and treat cancer for children.

**Results:**

The average availability of generics and original brands was 18.5% and 2.6%, respectively. In regions with different gross domestic product (GDP) per capita levels, the average availability was similar, but the city with lower GDP per capita levels had fewer tertiary public hospitals. The prices of most original brands were higher than the lowest-priced generics, and the median price ratios of 31 lowest-priced generics and 16 original brands were 0.744 (P25~P75, 0.446~2.791) and 2.908 (1.719~6.465). After paying medical insurance for medicines, the affordability of essential anticancer medicines was improved. The monthly medicine cost did not exceed 10% of the monthly household income for 78.9% (30/38) of the lowest-priced generics and 50.0% (8/16) of the original brands.

**Conclusion:**

The availability of lowest-priced generics was higher than original brands in public hospitals, but the availability of both was low, which was similar to previous studies in low- and middle-income countries. About half of the lowest-priced generics and 87.5% of the original brands cost more than 1.5 times the International Reference Price. Although the National Basic Medical Insurance greatly improved the affordability of essential anticancer medicines for children, higher subsidies for essential medicines for cancer treatment to limit catastrophic health expenditures are still recommended.

## Introduction

In recent years, the incidence of childhood cancer in low- and middle-income countries is still on the rise (1, 2). About 200,000 children worldwide are diagnosed with cancer every year (3), and approximately 80% of them live in low- and middle-income countries. Childhood cancer deaths in low- and middle-income countries account for more than 90% of global childhood cancer deaths (4). In China, cancer is the second leading cause of death in 5–14-years-old children (5), only slightly lower than the first leading cause of death-injury and poisoning. There are 25,000 new cases of childhood cancers under 15 years old and the incidence rate is increasing by 5% every year in China (6). The 5-year survival rate of childhood cancer remains low in low- and middle-income countries [ranging from 10 to 50% (3, 4)]. It is much lower than that in high-income countries, where the 5-year survival rate of childhood cancer has exceeded 80% (7–9).

Access to essential anticancer medicines is an indispensable component of pediatric cancer treatment, which affects the outcome of cancer and the children's prognosis. A key challenge to reducing the disparity in survival rates for childhood cancers between low- and middle-income countries (LMICs) and high-income countries (HICs) is to ensure access to reliable, high-quality, effective, and affordable essential anticancer medicines (10). In 2018, a global pediatric anticancer medicines survey showed that 42.9% of children in low- and middle-income countries had poor access to essential anticancer medicines, and 42.1% of children could not fully access standard chemotherapy regimens (11). It has been reported that less than 20% of children had access to anticancer therapy and afford it, and more than 80% of children were at risk of disease progression or even death due to unavailable or unaffordable treatment (12). In addition, the treatment cost of anticancer medicines is generally high. For example, according to a survey, the average treatment expense of pediatric acute lymphoblastic leukemia treated with chemotherapy alone ranged from 115,858 USD to 163,350 USD (13).

International organizations highly focus on access to anticancer medicines. In 2008, the Union for International Cancer Control (UICC) issued the World Cancer Declaration, which is committed to “access to accurate cancer diagnosis, appropriate cancer treatments, supportive care, rehabilitation services, and palliative care will have been improved for all patients with cancer worldwide” and “dramatically improving cancer survival rates in all countries” (14). In 2011, to improve access to essential anticancer medicines for children, the World Health Organization (WHO). Expert Committee advocated the inclusion of specific chemotherapy medicines for childhood cancers in the “Essential Medicines List for Children (EMLc)” (15). Then, with the active cooperation and efforts of the UICC, WHO, and International Society of Pediatric Oncology (SIOP). Working Group, more and more medicines recommended by the current pediatric oncology guidelines are further endorsed into the WHO EMLc, such as bleomycin, carboplatin, cisplatin, dacarbazine, etoposide, hydroxyurea, ifosfamide, and vincristine (16).

In China, like other low- and middle-income countries, to reduce the mortality and disease burden of childhood patients with cancer, it is strongly necessary to ensure access to anticancer medicines. However, the current study data on the accessibility of essential anticancer medicines for children are limited (17–19). Therefore, this study aims to assess the availability, price, and affordability of essential anticancer medicines for children in public hospitals in the Sichuan Province of China.

## Methods

The reporting of this study complied with the STROBE items (20).

### Study design

This study was a retrospective cross-sectional study on the accessibility of essential anticancer medicines for children in public hospitals in the Sichuan Province of China.

### Setting

Generally, tertiary hospitals are the first choice for patients to treat major diseases. So, we included all 152 tertiary public hospitals (120 general hospitals, 31 children's hospitals, and one cancer hospital) that could diagnose and treat cancer for children in Sichuan Province. The distribution of surveyed public hospitals and the gross domestic product (GDP) per capita level of each city in Sichuan province are shown in Supplementary Table 1. The GDP per capita level was based on the World Bank's standard classification of gross national income per capita in 2020.

### Participants

Because China did not have a National List of Essential Medicines, the surveyed medicines were selected by the comprehensive consideration of 2021 WHO EMLc (21), the “Additional Drugs for Advanced Care of Children With Cancer in Low- and Middle-Income Countries” (16) developed by the SIOP and WHO, the “2018 China National Essential Medicines List (NEML)” (22), the opinions of clinical anticancer pharmacists, and the listing of licensed medicines in China. Finally, all children's anticancer and related supportive medicines on the three lists and marketed in China, including 34 anticancer and three supportive essential medicines (a total of 97 specific strengths) for children were included in our study (Supplementary Table 2).

For each medicine, price and availability data were collected for two products: the originator brand (OB) and the lowest-priced generic (LPG). The OB was defined as a product marketed by the originator pharmaceutical company. The LPG was defined as the same efficacy product sold under the generic name with the lowest unit price at each public medicine outlet at the time of data collection in the survey.

### Variables

The WHO/HAI methodology was recommended assessing the accessibility of medicines by three indicators, including availability, price, and affordability.

The availability of medicines was judged by whether a medicine in a specific dosage form and strength was in stock in public hospitals, which was expressed as the percentage(%) of public hospitals that could provide medicines to all surveyed public hospitals (23).

Price was expressed by the median price ratios (MPRs), which were used to measure the price of essential anticancer medicines for children (23). The MPR was the ratio of the median unit price (the price of each tablet, capsule, vial, milliliter, gram, etc.) from our survey to the International Reference Price (IRP) (24). The IRPs for medicines were obtained from the median purchase prices in the International Drug Price Indicator Guide (DPIG) published by the Management Sciences for Health (MSH). If the median purchase prices were unobtainable, the median supplier prices were alternatives (24). When MPR ≤ 1.5, the price of the medicine was considered acceptable in public hospitals.


$$\begin{array}{l} MPR=\frac{Median\;unit\;price\;of\;medicines}{International\;reference\;price} \end{array}$$

According to the “Measuring medicine prices, availability, affordability and price components” published by WHO and Health Action International (HAI), the affordability of medicines was assessed by comparing the lowest-paid unskilled government worker's daily wage and medicines expenses in the standard treatment of children disease (for chronic diseases, the treatment course is 30 days). If the ratio was ≤1, it meant the medicine was affordable (23). Since the price of anticancer medicines was much higher than that of general medicines, this approach was considered inappropriate to assess the affordability of anticancer medicines. So this study used the catastrophic medicine expenditure indicator to assess the affordability of anticancer medicines (25, 26). When the medicine expenditure exceeded a certain proportion of the total household expenditure, it was considered that the family fell into “catastrophic expenditure” due to the payment of medical expenses (27, 28). In recent years, the most widely used thresholds are 10 and 25% (28, 29). In our study, the monthly household income replaced the monthly expenditure. When the medical expenses for a 30 day standard chemotherapy regimen did not exceed 10% of the monthly household income in Sichuan Province (25), the medicine was considered affordable; between 10% < and ≤ 25%, poor affordability; >25%, very poor affordability.


$$\begin{array}{l} Affordability=\frac{30-day\;medicine\;expenses}{Monthly\;household\;income} \end{array}$$

### Data sources

Based on the Sichuan Province Drug Use Monitoring Platform, the quantity and price of essential anticancer medicines in stock of surveyed public hospitals in 2020 were obtained. The data from this platform were officially collected by the Health Commission of Sichuan Province and included information on medicines in all medical institutions in Sichuan Province, which could reflect the actual situation and with good reliability. Moreover, the monthly household income in Sichuan Province came from the “2020 Chinese Health Statistics Yearbook” (30) (urban residents: 5 536.0 CNY, rural residents: 2 221.9 CNY, total residents: 3 743.4 CNY), and the exchange rate of 1 CNY = 0.1568 USD came from the State Administration of Foreign Exchange (31).

### Statistical methods

The data were organized and analyzed in Excel (32). In tables, we showed the availability of children's essential anticancer medicines included in the WHO EMLc (or not), in the NEML (or not), and at different reimbursement levels (Class A, Class B, and non-medical insurance medicines).

The median of MPR was used to assess the medicine price rationality, and the interquartile range (IQR) of the MPR was used to assess the dispersion.

Considering the incidence of childhood cancers in China (33), acute lymphoblastic leukemia, non-Hodgkin lymphoma, Hodgkin lymphoma, medulloblastoma, and nephroblastoma were selected to evaluate the medicine affordability. According to recommended doses and course of treatment of medicines in clinical guidelines, Uptodate (a clinical decision support database system), and medicine instructions (Supplementary Table 3), we calculated the 30 days' medicine treatment expenses for a child with a body surface area of 1 m^2^ and a weight of 30 kg (34). In addition, we calculated changes in medicine affordability after the National Basic Medical Insurance reimbursement. The reimbursable costs were equal to 60% of the costs of Class A medical insurance medicines or 60% of the 80% of costs of Class B medical insurance medicines, except for medicines specially stipulated by the National Basic Medical Insurance (60% was the reimbursement ratio of medical expenses in tertiary hospitals in Sichuan Province).

The chi-square test and the Wilcoxon rank sum test were used to compare the differences in average availability of essential anticancer medicines in different GDP per capita level regions and also compared the difference in the affordability of essential anticancer medicines between urban and rural residents before and after medical insurance reimbursing (The significance threshold was set at *p* < 0.05). Moreover, a comprehensive analysis of the availability and affordability was carried out through a four-quadrant diagram.

## Results

### Availability

The availability of 71 generics and 93 original brands was less than 30%, of which 37 generics and 76 original brands were not available. Only four generics had high availability (≥80%). Except for corticosteroids, the most available generic was 6 mg/ml paclitaxel injection (the availability was 82.9%), and the most available original brand was 200 mg cyclophosphamide injection powder for injection (the availability was 38.2%) (Supplementary Table 4).

The average availability of essential anticancer generics and original brands was 18.5 and 2.6%, respectively. Except for carboplatin, methylprednisolone, rituximab, and everolimus, the availability of generics was higher than the original brands. Moreover, for the 34 medicines not included in the NEML, the average availability of original brands and the generics was 0.3 and 7.3%, respectively. For the six medicines not covered by the National Basic Medical Insurance, the average availability of generics and original brands was 5.0 and 0.3%, respectively (Table 1).

**Table 1:** Average and median availability of essential anticancer medicines for children at the different types of public hospitals in Sichuan Province.

**Type**		**Average Availability (%)**				**Median Availability (%)**			
		**General Hospitals** **(*n =* 120)**	**Children's Hospital** **(*n =* 31)**	**Tumor Hospital** **(*n =* 1)**	**All public hospitals** **(*n =* 152)**	**General Hospitals** **(*n =* 120)**	**Children's Hospital** **(*n =* 31)**	**Tumor Hospital** **(*n =* 1)**	**All public hospitals** **(*n =* 152)**
**Originator brand**	WHO EMLc (*n =* 59)	1.9%	0.4%	11.9%	1.7%	0.0%	0.0%	0.0%	0.0%
	Non-WHO EMLc (*n =* 36)	4.9%	1.6%	5.6%	4.2%	0.0%	0.0%	0.0%	0.0%
	NEM (*n =* 61)	4.5%	1.3%	11.5%	3.9%	0.0%	0.0%	0.0%	0.0%
	Non-NEM (*n =* 34)	0.3%	0.1%	5.9%	0.3%	0.0%	0.0%	0.0%	0.0%
	Class A medical insurance (*n =* 53)	2.0%	1.0%	5.7%	1.8%	0.0%	0.0%	0.0%	0.0%
	Class B medical insurance (*n =* 36)	5.0%	0.9%	16.7%	4.3%	0.0%	0.0%	0.0%	0.0%
	Non-medical insurance (*n =* 6)	0.0%	0.0%	0.0%	0.0%	0.0%	0.0%	0.0%	0.0%
	Total (*n =* 95)	3.0%	0.9%	9.5%	2.6%	0.0%	0.0%	0.0%	0.0%
**Generic**	WHO EMLc (*n =* 59)	19.2%	2.6%	30.5%	15.9%	0.8%	0.0%	0.0%	1.3%
	Non-WHO EMLc (*n =* 36)	24.5%	15.4%	33.3%	22.7%	8.7%	4.8%	0.0%	7.9%
	NEM (*n =* 61)	28.1%	11.2%	39.3%	24.7%	14.2%	3.2%	0.0%	12.5%
	Non-NEM (*n =* 34)	8.9%	0.9%	17.6%	7.3%	0.0%	0.0%	0.0%	0.0%
	Class A medical insurance (*n =* 53)	24.6%	10.7%	35.8%	21.8%	6.7%	3.2%	0.0%	7.2%
	Class B medical insurance (*n =* 36)	18.8%	3.8%	27.8%	15.8%	10.0%	0.0%	0.0%	8.9%
	Non-medical insurance (*n =* 6)	6.1%	0.5%	16.7%	5.0%	0.0%	0.0%	0.0%	0.0%
	Total (*n =* 95)	21.2%	7.5%	31.6%	18.5%	5.0%	0.0%	0.0%	5.9%

WHO EMLc, medicines on 2021 WHO Essential Medicines List for Children; Non-WHO EMLc, medicines not on 2021 WHO Essential Medicines List for Children; NEM, medicines on the 2018 China National Essential Medicines List; Non-NEM, medicines not on the 2018 China National Essential Medicines List; Class A Medical Insurance, medicines classified as Class A reimbursement by the Basic Medical Insurance; Class B Medical Insurance, medicines classified as Class B reimbursement by the Basic Medical Insurance; Non-Medical Insurance, medicines classified as not reimbursed by the Basic Medical Insurance.

In general hospitals, the average availability of original brands and generics was 3.0 and 21.2%, respectively. In children's hospitals, the average availability of original brands and generics was 0.9 and 7.5%, respectively. In the tumor hospital, the average availability of original brands and generics was 9.5 and 31.6%. The average availability of essential anticancer medicines for children in the tumor hospital was higher than in general hospitals and children's hospitals (Table 1).

**Figure 1:**
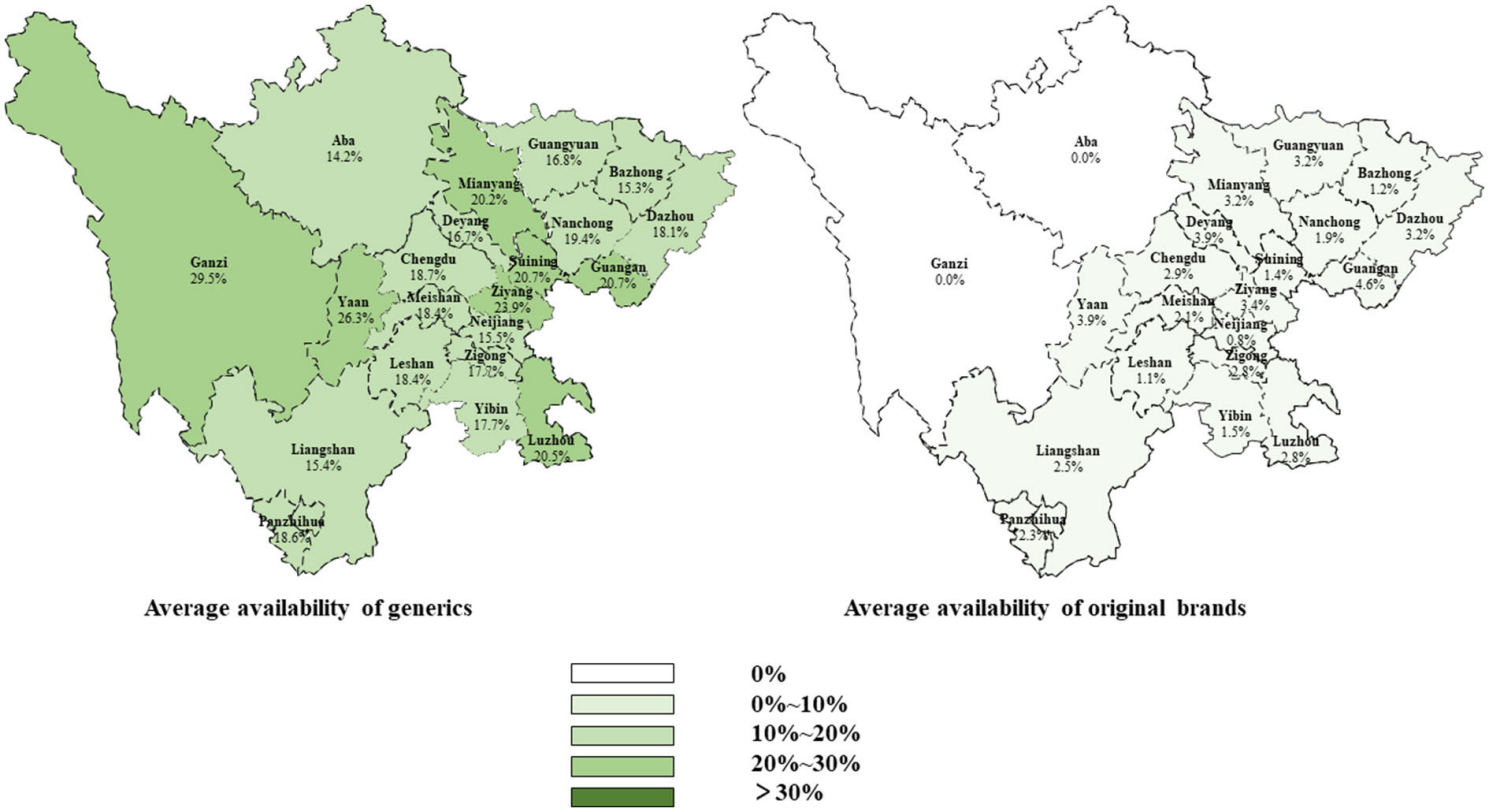
Average availability of originator brands and generics in different cities of Sichuan Province.

Moreover, the average availability of generics and OBs in different cities was similar at less than 30% (Figure 1). The average availability of essential anticancer medicines for children in regions with different GDP per capita levels was similar, but the city with lower GDP per capita levels had fewer tertiary public hospitals to diagnose and treat childhood cancer (Supplementary Table 5).

### Price

Only 58 lowest-priced generics and 19 original brands of 97 surveyed products could be available in public hospitals, of which the international reference price (IPR) of 27 lowest-priced generics and three original brands were not obtained, so the MPR could not be calculated. The MPR of the 31 lowest-priced generics was 0.744 (P25~P75, 0.446~2.791), and the 16 original brands were 2.908 (P25~P75, 1.719~6.465). Moreover, 41.9% (13/31) of lowest-priced generics and 87.5% (14/16) of original brands had MPR > 1.5, which indicated that these medicines' prices were too expensive. For generics, the 40 mg/2 ml etoposide injection had the highest MPR of 64.039. For original brands, the 100 mg imatinib tablet had the highest MPR of 38.916 (Table 2).

**Table 2:** Prices of essential anticancer medicines for children in the public hospitals in Sichuan Province.

**Medicine generic Name**	**Dosage form**	**Strength**	**IRP in 2015 (USD)**	**Adjusted IRP (USD)**	**Lowest-priced generics**		**Original brands**	
					**Unit price**	**MPR**	**Unit price**	**MPR**
					**(USD)**		**(USD)**	
Doxorubicin	Powder for injection	10 mg	2.1232/vial	1.9153/vial	3.503/vial	1.829	3.506/vial	1.831
Cytarabine	Powder for injection	100 mg	3.4774/vial	3.2407/vial	2.412/vial	0.744	5.802/vial	1.790
Cytarabine	Powder for injection	50 mg	—	—	1.176/vial	—	—	—
Oxaliplatin	Powder for injection	50 mg	28.8821/vial	9.1951/vial	6.020/vial	0.655	—	—
Oxaliplatin	Powder for injection	100 mg	74.7676/vial	26.1018/vial	15.388/vial	0.590	—	—
Allopurinol	Tablet	100 mg	0.0236/tab	0.0309/tab	0.141/tab	4.563	—	—
Bleomycin	Powder for injection	15 mg	12.3210/vial	25.767/vial	18.659/vial	0.724	21.717/vial	0.843
Dacarbazine	Powder for injection	100 mg	—	—	8.765/vial	—	—	—
Dasatinib	Tablet	20 mg	—	—	0.986/tab	—	—	—
Dasatinib	Tablet	50 mg	—	—	8.447/tab	—	19.077/tab	—
Dexamethasone	Tablet	0.75 mg	—	—	0.007/tab	—	—	—
Dexamethasone	Injection	2 mg/1ml	—	—	0.020/ml	—	—	—
Dexamethasone	Injection	5 mg/1ml	0.0827/ml	0.0668/ml	0.049/ml	0.734	—	—
Fluorouracil	Injection	0.25 g/10ml	0.2600/ml	0.3647/ml	0.823/ml	2.257	—	—
Cyclophosphamide	Powder for injection	200 mg	2.0838/vial	1.5293/vial	4.152/vial	2.715	3.787/vial	2.476
Methotrexate	Tablet	2.5 mg	0.0629/tab	0.0583/tab	0.381/tab	6.535	—	—
Methotrexate	Powder for injection	50 mg	—	—	7.809/vial	—	—	—
Methotrexate	Powder for injection	5 mg	—	—	0.347/vial	—	—	—
Methotrexate	Powder for injection	100 mg	—	—	1.880/vial	—	—	—
Methylprednisolone	Tablet	4 mg	—	—	0.169/tab	—	0.153/tab	—
Methylprednisolone	Powder for injection	40 mg	1.3505/vial	1.0146/vial	2.327/vial	2.294	3.704/vial	3.651
Methylprednisolone	Powder for injection	500 mg	5.8357/vial	5.9061/vial	2.708/vial	0.459	19.730/vial	3.341
Carboplatin	Injection	150 mg/15 ml	16.0053/vial	18.339/vial	—	—	24.553/vial	1.339
Carboplatin	Powder for injection	50 mg	—	—	1.328/vial	—	—	—
Carboplatin	Powder for injection	100 mg	—	—	4.149/vial	—	—	—
Rituximab	Injection	100 mg/10 ml	13.6721/ml	6.663/ml	—	—	35.977/ml	5.400
Rituximab	Injection	500 mg/50 ml	13.6721/ml	6.663/ml	—	—	24.669/ml	3.702
Mesna	Injection	400 mg/4 ml	0.7345/ml	1.2068/ml	0.361/ml	0.299	—	—
Asparaginase	Powder for injection	10,000 IU/vial	52.8846/vial	77.9561/vial	17.905/vial	0.230	—	—
Asparaginase	Powder for injection	5,000 IU/vial	—	—	18.910/vial	—	—	—
Pegaspargase	Injection	3,750 IU/5 ml	—	—	467.264/vial	—	—	—
Bleomycin A5	Powder for injection	8 mg	—	—	125.126/vial	—	—	—
Hydroxycarbamide	Tablet	500 mg	0.2174/tab	0.1773/tab	0.107/tab	0.603	—	—
Hydrocortisone	Powder for injection	100 mg	0.5200/vial	0.5242/vial	1.444/vial	2.755	—	—
Hydrocortisone	Tablet	20 mg	0.0640/tab	0.0495/tab	0.140/tab	2.828	—	—
Hydrocortisone	Injection	10 mg/2 ml	—	—	0.023/vial	—	—	—
Hydrocortisone	Injection	25 mg/5 ml	—	—	0.131/vial	—	—	—
Hydrocortisone	Injection	100 mg/20 ml	—	—	0.002/vial	—	—	—
Hydrocortisone	Powder for injection	50 mg	—	—	0.655/vial	—	—	—
Mercaptopurine	Tablet	50 mg	2.2360/tab	3.5056/tab	0.259/tab	0.074	—	—
All-trans retinoid acid (ATRA)	Capsule	10 mg	—	—	0.256/cap	—	—	—
Daunorubicin	Powder for injection	20 mg	19.3247/vial	12.5708/vial	3.889/vial	0.309	4.215/vial	0.335
Arsenic trioxide	Injection	1 mg/ml	—	—	18.816/ml	—	—	—
Arsenic trioxide	Powder for injection	10 mg	—	—	21.134/vial	—	—	—
Calcium folinate	Tablet	15 mg	1.2953/tab	1.1649/tab	0.380/tab	0.326	—	—
Calcium folinate	Injection	100 mg/10 ml	—	—	0.393/vial	—	—	—
Calcium folinate	Powder for injection	25 mg	—	—	15.994/vial	—	—	—
Calcium folinate	Powder for injection	50 mg	2.3419/vial	1.6717/vial	0.956/vial	0.572	—	—
Calcium folinate	Powder for injection	100 mg	—	—	1.182/vial	—	—	—
Irinotecan	Injection	40 mg/2 ml	5.7777/ml	5.7777/ml	24.836/ml	4.299	81.536/ml	14.112
Irinotecan	Injection	100 mg/5 ml	5.7777/ml	5.7777/ml	20.035/ml	3.468	55.821/ml	9.661
Imatinib	Tablet	100 mg	0.6932/tab	0.4823/tab	1.625/tab	3.369	18.769/tab	38.916
Etoposide	Injection	100 mg/5 mL	0.4036/ml	0.3085/ml	0.244/ml	0.791	—	—
Etoposide	Capsule	50 mg	—	—	1.975/cap	—	—	—
Etoposide	Injection	40 mg/2 ml	0.4036/ml	0.3085/ml	19.756/ml	64.039	—	—
Everolimus	Tablet	5 mg	—	—	—	—	20.384/tab	—
Ifosfamide	Powder for injection	500 mg	21.5481/vial	14.1326/vial	6.131/vial	0.434	—	—
Ifosfamide	Powder for injection	1 g	26.7130/vial	21.3422/vial	6.264/vial	0.294	32.113/vial	1.505
Vinorelbine	Capsule	20 mg	—	—	21.952/cap	—	—	—
Vinorelbine	Injection	10 mg/ml	21.9650/vial	21.965/vial	8.748/vial	0.398	44.453/vial	2.024
Vincristine	Powder for injection	1 mg	2.5416/vial	2.4912/vial	30.576/vial	12.274	—	—
Paclitaxel	Injection	6 mg/ml	0.8754/ml	0.6276/ml	0.834/ml	1.329	16.026/ml	25.535

### Affordability

We evaluated the affordability of commonly used essential anticancer medicines (a total of 39 medicines, including 38 available lowest-priced generics and 16 available original brands) for five common cancers or tumors in children, including acute lymphoblastic leukemia, non-Hodgkin lymphoma, Hodgkin lymphoma, medulloblastoma, and nephroblastoma. Before medical insurance reimbursement, among the 38 lowest-priced generics, 24 lowest-priced generics had good affordability and the 30-day medicine expenses did not exceed 10% of the monthly household income. And six lowest-priced generics had poor affordability and the monthly medicine expenses accounted for 10 to 25% of monthly household income. Eight lowest-priced generics had been very poor and exceeded 25% of monthly household income (Table 3). Among 16 original brands, seven original brands had good affordability, three original brands had poor affordability, and six original brands had very poor affordability (Table 4). Moreover, there were 26 lowest-priced generics and seven original brands that could be afforded by urban residents, 22 and three by rural residents. There was no significant difference in the affordability of essential anticancer medicines for children in public hospitals between urban and rural residents (lowest-priced generics, *P* = 0.307; original brands, *P* = 0.173).

**Table 3:** Affordability of lowest-price generics before and after medical insurance reimbursement.

**Disease**	**Medicine**	**Before Medical Insurance Reimbursement**				**After Medical Insurance Reimbursement**			
		**30d Medicine**	**30d Medicine Expenses/**			**30d Medicine**	**30d Medicine Expenses/**		
		**Expenses (USD)**	**Monthly Household Income (%)**			**Expenses (USD)**	**Monthly Household Income (%)**		
			**Urban Residents** **(868.04 USD)**	**Rural Residents** **(348.39 USD)**	**All Residents** **(586.97 USD)**		**Urban Residents** **(868.04 USD)**	**Rural Residents** **(348.39 USD)**	**All Residents** **(586.97 USD)**
Acute lymphoblastic leukemia	Asparaginase, powder for injection, 10,000 IU/vial	268.57	30.9%	77.1%	45.8%	107.43	12.4%	30.8%	18.3%
	Asparaginase, powder for injection, 5,000 IU/vial	537.15	61.9%	154.2%	91.5%	214.86	24.8%	61.7%	36.6%
	Cyclophosphamide, powder for injection, 200 mg	18.68	2.2%	5.4%	3.2%	7.47	0.9%	2.1%	1.3%
	Cytarabine, powder for injection, 50 mg	23.52	2.7%	6.8%	4.0%	9.41	1.1%	2.7%	1.6%
	Cytarabine, powder for injection, 100 mg	24.12	2.8%	6.9%	4.1%	9.65	1.1%	2.8%	1.6%
	Daunorubici, powder for injection, 20 mg	26.25	3.0%	7.5%	4.5%	10.50	1.2%	3.0%	1.8%
	Dexamethasone, tablet, 0.75 mg	2.43	0.3%	0.7%	0.4%	0.97	0.1%	0.3%	0.2%
	Dexamethasone, injection, 2 mg/1 ml	2.65	0.3%	0.8%	0.5%	1.06	0.1%	0.3%	0.2%
	Dexamethasone, injection, 5 mg /1 ml	2.62	0.3%	0.8%	0.4%	1.05	0.1%	0.3%	0.2%
	Doxorubicin, powder for injection, 10 mg	52.54	6.1%	15.1%	9.0%	21.02	2.4%	6.0%	3.6%
	Etoposide, injection, 40 mg /2 ml	493.90	56.9%	141.8%	84.1%	197.56	22.8%	56.7%	33.7%
	Etoposide, injection, 100 mg /5 ml	6.11	0.7%	1.8%	1.0%	2.44	0.3%	0.7%	0.4%
	Hydrocortisone, injection, 10 mg /2 ml	1.71	0.2%	0.5%	0.3%	0.68	0.1%	0.2%	0.1%
	Hydrocortisone, injection, 25 mg /5 ml	3.93	0.5%	1.1%	0.7%	1.57	0.2%	0.5%	0.3%
	Hydrocortisone, injection, 100 mg /20 ml	0.02	0.0%	0.0%	0.0%	0.01	0.0%	0.0%	0.0%
	Hydrocortisone, powder for injection, 50 mg	9.83	1.1%	2.8%	1.7%	3.93	0.5%	1.1%	0.7%
	Hydrocortisone, powder for injection, 100 mg	10.83	1.2%	3.1%	1.8%	4.33	0.5%	1.2%	0.7%
	Hydrocortisone, tablet, 20 mg	5.26	0.6%	1.5%	0.9%	2.10	0.2%	0.6%	0.4%
	Imatinib, tablet, 100 mg	292.54	33.7%	84.0%	49.8%	152.12	17.5%	43.7%	25.9%
	Mercaptopurine, tablet, 50 mg	15.52	1.8%	4.5%	2.6%	6.21	0.7%	1.8%	1.1%
	Methotrexate, tablet, 2.5 mg	18.30	2.1%	5.3%	3.1%	7.32	0.8%	2.1%	1.2%
	Methotrexate, powder for injection, 5 mg	8.32	1.0%	2.4%	1.4%	3.33	0.4%	1.0%	0.6%
	Methotrexate, powder for injection, 50 mg	18.74	2.2%	5.4%	3.2%	7.50	0.9%	2.2%	1.3%
	Methotrexate, powder for injection, 100 mg	2.26	0.3%	0.6%	0.4%	0.90	0.1%	0.3%	0.2%
	Methylprednisolone, tablet, 4 mg	60.92	7.0%	17.5%	10.4%	24.37	2.8%	7.0%	4.2%
	Methylprednisolone, powder for injection, 40 mg	83.77	9.7%	24.0%	14.3%	43.56	5.0%	12.5%	7.4%
	Methylprednisolone, powder for injection 500 mg	7.80	0.9%	2.2%	1.3%	4.06	0.5%	1.2%	0.7%
	Pegaspargase, injection, 3,750 IU/ 5ml	623.02	71.8%	178.8%	106.1%	249.21	28.7%	71.5%	42.5%
	Vincristine, powder for injection, 1 mg	275.18	31.7%	79.0%	46.9%	110.07	12.7%	31.6%	18.8%
Non-Hodgkin lymphoma	Cyclophosphamide, powder for injection, 200 mg	18.68	2.2%	5.4%	3.2%	7.47	0.9%	2.1%	1.3%
	Cytarabine, powder for injection, 100 mg	24.12	2.8%	6.9%	4.1%	9.65	1.1%	2.8%	1.6%
	Dexamethasone, tablet, 0.75 mg	2.43	0.3%	0.7%	0.4%	0.97	0.1%	0.3%	0.2%
	Dexamethasone, injection, 2 mg/ 1 ml	2.65	0.3%	0.8%	0.5%	1.06	0.1%	0.3%	0.2%
	Dexamethasone, injection, 5 mg/ 1 ml	2.62	0.3%	0.8%	0.4%	1.05	0.1%	0.3%	0.2%
	Doxorubicin, powder for injection, 10 mg	52.54	6.1%	15.1%	9.0%	21.02	2.4%	6.0%	3.6%
	Etoposide, injection, 40 mg /2 ml	296.34	34.1%	85.1%	50.5%	118.54	13.7%	34.0%	20.2%
	Etoposide, injection, 100 mg /5 ml	3.66	0.4%	1.1%	0.6%	1.47	0.2%	0.4%	0.2%
	Hydrocortisone, injection, 10 mg /2 ml	1.71	0.2%	0.5%	0.3%	0.68	0.1%	0.2%	0.1%
	Hydrocortisone, injection, 25 mg /5 ml	3.93	0.5%	1.1%	0.7%	1.57	0.2%	0.5%	0.3%
	Hydrocortisone, injection, 100 mg /20 ml	0.02	0.0%	0.0%	0.0%	0.01	0.0%	0.0%	0.0%
	Hydrocortisone, powder for injection, 50 mg	9.83	1.1%	2.8%	1.7%	3.93	0.5%	1.1%	0.7%
	Hydrocortisone, powder for injection, 100 mg	10.83	1.2%	3.1%	1.8%	4.33	0.5%	1.2%	0.7%
	Hydrocortisone, tablet, 20 mg	5.26	0.6%	1.5%	0.9%	2.10	0.2%	0.6%	0.4%
	Ifosfamide, powder for injection, 500 mg	110.36	12.7%	31.7%	18.8%	57.39	6.6%	16.5%	9.8%
	Ifosfamide, powder for injection, 1 g	56.38	6.5%	16.2%	9.6%	29.32	3.4%	8.4%	5.0%
	Mesna, injection, 400 mg /4 ml	90.36	10.4%	25.9%	15.4%	46.99	5.4%	13.5%	8.0%
	Methotrexate, tablet, 2.5 mg	18.30	2.1%	5.3%	3.1%	7.32	0.8%	2.1%	1.2%
	methotrexate, powder for injection, 5 mg	8.32	1.0%	2.4%	1.4%	3.33	0.4%	1.0%	0.6%
	Methotrexate, powder for injection, 50 mg	18.74	2.2%	5.4%	3.2%	7.50	0.9%	2.2%	1.3%
	Methotrexate, powder for injection, 100 mg	2.26	0.3%	0.6%	0.4%	0.90	0.1%	0.3%	0.2%
	Methylprednisolone, tablet, 4 mg	60.92	7.0%	17.5%	10.4%	24.37	2.8%	7.0%	4.2%
	Methylprednisolone, powder for injection, 40 mg	83.77	9.7%	24.0%	14.3%	43.56	5.0%	12.5%	7.4%
	Methylprednisolone, powder for injection, 500 mg	7.80	0.9%	2.2%	1.3%	4.06	0.5%	1.2%	0.7%
	Vincristine, powder for injection, 1 mg	275.18	31.7%	79.0%	46.9%	110.07	12.7%	31.6%	18.8%
Hodgkin lymphoma	Bleomycin, powder for injection, 15 mg	99.52	11.5%	28.6%	17.0%	51.75	6.0%	14.9%	8.8%
	Cyclophosphamide, powder for injection, 200 mg	18.68	2.2%	5.4%	3.2%	7.47	0.9%	2.1%	1.3%
	Ifosfamide, powder for injection, 500 mg	110.36	12.7%	31.7%	18.8%	57.39	6.6%	16.5%	9.8%
	Ifosfamide, powder for injection, 1 g	56.38	6.5%	16.2%	9.6%	29.32	3.4%	8.4%	5.0%
	Mesna, injection, 400 mg /4 ml	90.36	10.4%	25.9%	15.4%	46.99	5.4%	13.5%	8.0%
	Dacarbazine, powder for injection, 100 mg	109.56	12.6%	31.4%	18.7%	56.97	6.6%	16.4%	9.7%
	Doxorubicin, powder for injection, 10 mg	52.54	6.1%	15.1%	9.0%	21.02	2.4%	6.0%	3.6%
	Etoposide, injection, 40 mg /2 ml	493.90	56.9%	141.8%	84.1%	197.56	22.8%	56.7%	33.7%
	Etoposide, injection, 100 mg /5 ml	6.11	0.7%	1.8%	1.0%	2.44	0.3%	0.7%	0.4%
	Vincristine, powder for injection, 1 mg	275.18	31.7%	79.0%	46.9%	110.07	12.7%	31.6%	18.8%
Medulloblastoma	Carboplatin, powder for injection, 50 mg	14.87	1.7%	4.3%	2.5%	5.95	0.7%	1.7%	1.0%
	Carboplatin, powder for injection, 100 mg	23.23	2.7%	6.7%	4.0%	9.29	1.1%	2.7%	1.6%
	Vincristine, powder for injection, 1 mg	275.18	31.7%	79.0%	46.9%	110.07	12.7%	31.6%	18.8%
	Etoposide, injection, 40 mg /2 ml	493.90	56.9%	141.8%	84.1%	197.56	22.8%	56.7%	33.7%
	Etoposide, injection, 100 mg /5 ml	6.11	0.7%	1.8%	1.0%	2.44	0.3%	0.7%	0.4%
	Methotrexate, tablet, 2.5 mg	18.30	2.1%	5.3%	3.1%	7.32	0.8%	2.1%	1.2%
	Methotrexate, powder for injection, 5 mg	8.32	1.0%	2.4%	1.4%	3.33	0.4%	1.0%	0.6%
	Methotrexate, powder for injection, 50 mg	18.74	2.2%	5.4%	3.2%	7.50	0.9%	2.2%	1.3%
	Methotrexate, powder for injection, 100 mg	2.26	0.3%	0.6%	0.4%	0.90	0.1%	0.3%	0.2%
	Cyclophosphamide, powder for injection, 200 mg	18.68	2.2%	5.4%	3.2%	7.47	0.9%	2.1%	1.3%
Nephroblastoma	Carboplatin, powder for injection, 50 mg	21.25	2.4%	6.1%	3.6%	8.50	1.0%	2.4%	1.4%
	Carboplatin, powder for injection, 100 mg	33.19	3.8%	9.5%	5.7%	13.28	1.5%	3.8%	2.3%
	Vincristine, powder for injection, 1 mg	275.18	31.7%	79.0%	46.9%	110.07	12.7%	31.6%	18.8%
	Doxorubicin, powder for injection, 10 mg	52.54	6.1%	15.1%	9.0%	21.02	2.4%	6.0%	3.6%
	Etoposide, injection, 40 mg /2 ml	296.34	34.1%	85.1%	50.5%	118.54	13.7%	34.0%	20.2%
	Etoposide, injection, 100 mg /5 ml	3.66	0.4%	1.1%	0.6%	1.47	0.2%	0.4%	0.2%
	Cyclophosphamide, powder for injection, 200 mg	18.68	2.2%	5.4%	3.2%	7.47	0.9%	2.1%	1.3%
	Ifosfamide, powder for injection, 500 mg	110.36	12.7%	31.7%	18.8%	57.39	6.6%	16.5%	9.8%
	Ifosfamide, powder for injection, 1 g	56.38	6.5%	16.2%	9.6%	29.32	3.4%	8.4%	5.0%
	Irinotecan, injection, 40 mg /2 ml	397.38	45.8%	114.1%	67.7%	206.64	23.8%	59.3%	35.2%
	Irinotecan, injection, 100 mg /5 ml	320.56	36.9%	92.0%	54.6%	166.69	19.2%	47.8%	28.4%
	Mesna, injection, 400 mg /4 ml	90.36	10.4%	25.9%	15.4%	46.99	5.4%	13.5%	8.0%

**Table 4:** Affordability of original brands before and after medical insurance reimbursement.

**Disease**	**Medicine**	**Before Medical Insurance Reimbursement**				**After Medical Insurance Reimbursement**			
		**30d Medicine**	**30d Medicine Expenses/**			**30d Medicine**	**30d Medicine Expenses/**		
		**Expenses (USD)**	**Monthly Household Income (%)**			**Expenses (USD)**	**Monthly Household Income (%)**		
			**Urban Residents** **(868.04 USD)**	**Rural Residents** **(348.39 USD)**	**All Residents** **(586.97 USD)**		**Urban Residents** **(868.04 USD)**	**Rural Residents** **(348.39 USD)**	**All Residents** **(586.97 USD)**
Acute lymphoblastic leukemia	Cyclophosphamide, powder for injection, 200 mg	17.04	2.0%	4.9%	2.9%	6.82	0.8%	2.0%	1.2%
	Cytarabine, powder for injection, 100 mg	58.02	6.7%	16.7%	9.9%	23.21	2.7%	6.7%	4.0%
	Daunorubicin, powder for injection, 20 mg	28.45	3.3%	8.2%	4.8%	11.38	1.3%	3.3%	1.9%
	Doxorubicin, powder for injection, 10 mg	52.59	6.1%	15.1%	9.0%	21.04	2.4%	6.0%	3.6%
	Imatinib, tablet, 100 mg	3,378.41	389.2%	969.7%	575.6%	1,756.77	202.4%	504.2%	299.3%
	Methylprednisolone, tablet, 4 mg	54.91	6.3%	15.8%	9.4%	21.96	2.5%	6.3%	3.7%
	Methylprednisolone, powder for injection, 40 mg	133.33	15.4%	38.3%	22.7%	69.33	8.0%	19.9%	11.8%
	Methylprednisolone, powder for injection, 500 mg	56.82	6.5%	16.3%	9.7%	29.55	3.4%	8.5%	5.0%
Non-Hodgkin lymphoma	Cyclophosphamide, powder for injection, 200 mg	17.04	2.0%	4.9%	2.9%	6.82	0.8%	2.0%	1.2%
	Cytarabine, powder for injection, 50 mg	23.52	2.7%	6.8%	4.0%	9.41	1.1%	2.7%	1.6%
	Cytarabine, powder for injection, 100 mg	58.02	6.7%	16.7%	9.9%	23.21	2.7%	6.7%	4.0%
	Doxorubicin, powder for injection, 10 mg	52.59	6.1%	15.1%	9.0%	21.04	2.4%	6.0%	3.6%
	Ifosfamide, powder for injection, 1 g	289.01	33.3%	83.0%	49.2%	150.29	17.3%	43.1%	25.6%
	Methylprednisolon, tablet, 4 mg	54.91	6.3%	15.8%	9.4%	21.96	2.5%	6.3%	3.7%
	Methylprednisolone, powder for injection, 40 mg	133.33	15.4%	38.3%	22.7%	69.33	8.0%	19.9%	11.8%
	Methylprednisolone, powder for injection, 500 mg	56.82	6.5%	16.3%	9.7%	29.55	3.4%	8.5%	5.0%
	Rituximab, injection, 100 mg/10 ml	2,698.26	310.8%	774.5%	459.7%	1,403.10	161.6%	402.7%	239.0%
	Rituximab, injection, 500 mg/50 ml	1,850.14	213.1%	531.0%	315.2%	962.08	110.8%	276.1%	163.9%
Hodgkin lymphoma	Bleomycin, powder for injection, 15 mg	115.82	13.3%	33.2%	19.7%	60.23	6.9%	17.3%	10.3%
	Cyclophosphamide, powder for injection, 200 mg	17.04	2.0%	4.9%	2.9%	6.82	0.8%	2.0%	1.2%
	Ifosfamide, powder for injection, 1 g	289.01	33.3%	83.0%	49.2%	150.29	17.3%	43.1%	25.6%
	Doxorubicin, powder for injection, 10 mg	52.59	6.1%	15.1%	9.0%	21.04	2.4%	6.0%	3.6%
Medulloblastoma	Carboplatin, injection, 150 mg/15 ml	91.67	10.6%	26.3%	15.6%	36.67	4.2%	10.5%	6.2%
	Cyclophosphamide, powder for injection, 200 mg	17.04	2.0%	4.9%	2.9%	6.82	0.8%	2.0%	1.2%
Nephroblastoma	Carboplatin, injection, 150 mg/15 ml	130.95	15.1%	37.6%	22.3%	52.38	6.0%	15.0%	8.9%
	Doxorubicin, powder for injection, 10 mg	52.59	6.1%	15.1%	9.0%	21.04	2.4%	6.0%	3.6%
	Cyclophosphamide, powder for injection, 200 mg	17.04	2.0%	4.9%	2.9%	6.82	0.8%	2.0%	1.2%
	Ifosfamide, powder for injection, 1 g	289.01	33.3%	83.0%	49.2%	150.29	17.3%	43.1%	25.6%
	Irinotecan, injection, 40 mg/2 ml	1,304.58	150.3%	374.5%	222.3%	678.38	78.2%	194.7%	115.6%
	Irinotecan, injection, 100 mg/5 ml	893.13	102.9%	256.4%	152.2%	464.43	53.5%	133.3%	79.1%

After medical insurance reimbursement, the affordability of essential anticancer medicines for children was improved. For lowest-priced generics, the number of affordable medicines increased to 30, and the number of medicines with very poor affordability decreased to 2. For original brands, the number of affordable medicines increased to 8, and the number of poorly affordable medicines decreased to 2 (Supplementary Table 6).

Before the medical insurance reimbursement, the lowest-priced generics and original brands with the worst affordability were pegaspargase (injection, 3,750 IU/ 5 ml) and imatinib (tablet, 100 mg), and the 30-day medicine expenses were about 106.1 and 575.6% of the monthly household income of residents. But the medicine expenses dropped to 42.5 and 299.3% of the monthly household income after medical insurance reimbursement.

### Comprehensive comparison of availability and affordability

**Figure 2:**
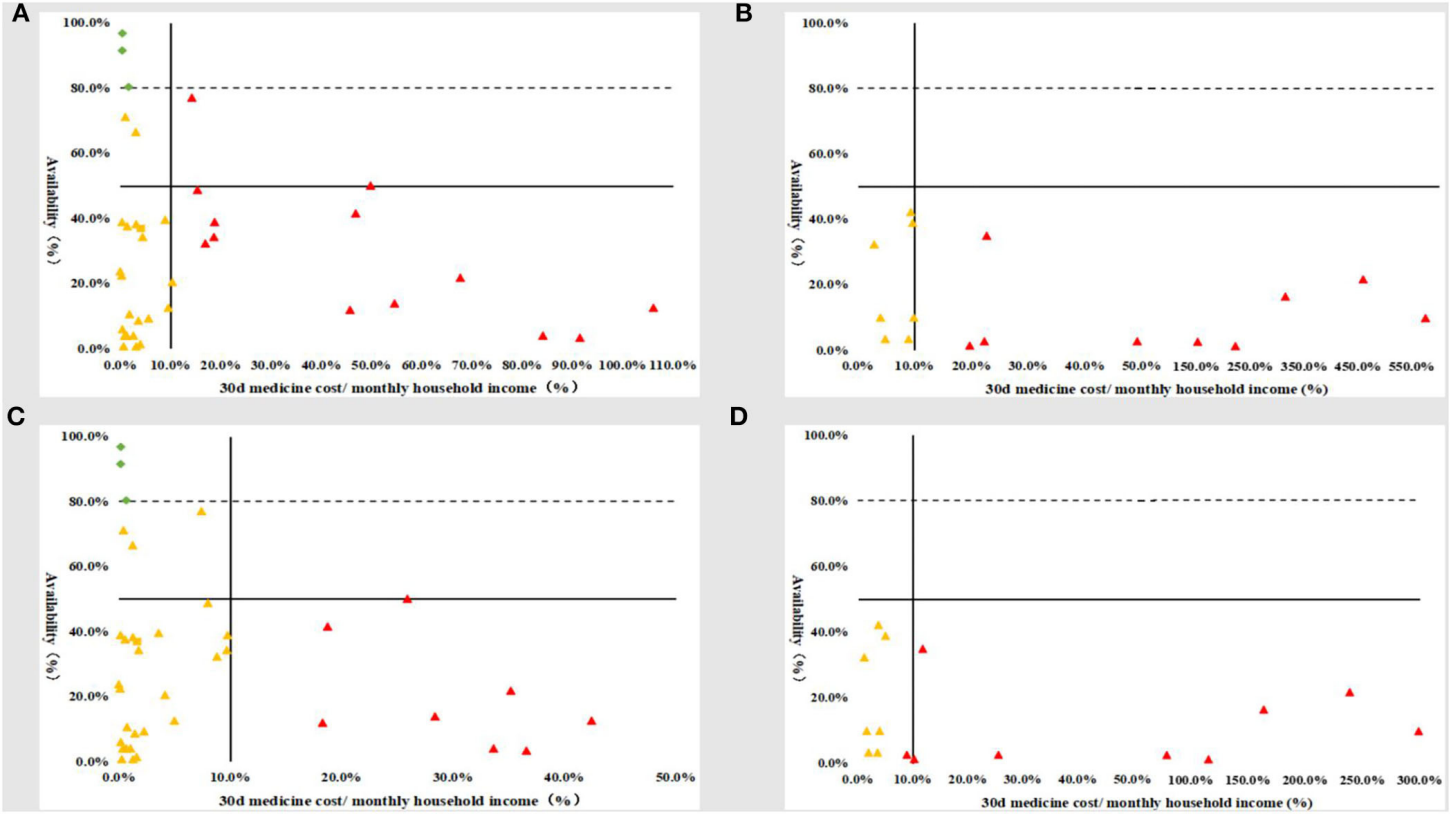
Comprehensive comparison of the availability and affordability of children's essential anticancer medicines in the public hospitals in Sichuan Province. **(A)** Before medical insurance reimbursement_generics; **(B)** before medical insurance reimbursement_original brands; **(C)** after medical insurance reimbursement_generics; **(D)** after medical insurance reimbursement_original brands.

Only three generics and 0 original brands simultaneously had availability ≥80% and good affordability (30 days medicine expenses ≤10% of monthly household income). Most affordable medicines were poorly available: before medical insurance reimbursement, 21 generics (55.3%) and seven original brands (43.8%); after medical insurance reimbursement, 27 generics (71.1%) and eight original brands (50.0%). Moreover, there were many medicines with poor affordability and poor availability: before medical insurance reimbursement, 14 generics (36.8%) and nine original brands (56.3%); after medical insurance reimbursement, eight generics (21.1%) and eight original brands (50.0%) (Figure 2).

## Discussion

The study results showed that 88.7% (86/97) generics and all original brands could be available in less than 50% of surveyed public hospitals in Sichuan Province, including cyclophosphamide (35), methotrexate (36), daunorubicin (37), and mercaptopurine (38), which were commonly used, inexpensive and affordable medicines. Vassal et al. (17) investigated the availability of 36 essential anticancer medicines for children and adolescents in European countries. The results showed that the median availability of 24 essential anticancer medicines included in WHO EMLc was 92%, and the median availability of 44 not included was 73%. Only 13.24% (9/68) of essential anticancer medicines for children and adolescents were available in less than 50% of institutions. Most of them were biological preparations and liposomes which were relatively expensive (e.g., doxorubicin liposomal, cytarabine liposomal, etoposide phosphate, dinutuximab, blinatumomab, prednisone oral liquid, etc.). Some researchers compared the availability of children's essential anticancer medicines in different income-level countries and found that the average availability in low- and middle-income countries was low at 13.6% (median availability was 12.5%). And due to the poor stability of the medicine supply chain, low- and middle-income countries were prone to children's essential anticancer medicine shortages (19). At present, there was no study on the availability of children's essential anticancer medicines in China. Compared with results in Chinese adults (39), we found that the availability of essential anticancer medicines for adults was slightly higher than for children, but still at a lower level. The average availability of essential anticancer original brands for adults in public hospitals was 16.9% (median availability was 5.9%) and 29.9% (median availability was 29.4%) for generics. In recent years, the Chinese government has attached great importance to the medicines shortage. In 2020, the “National Key Monitoring List of Clinically Necessary and Shortage-Prone Medicines” (40) was issued by the National Health Commission of the People's Republic of China, which included a number of anticancer medicines, such as methotrexate injection, hydrocortisone injection, cyclophosphamide injection, mercaptopurine tablet, cytarabine injection, bleomycin injection, vincristine injection, and tretinoin tablets. Our findings also showed the low availability of those anticancer medicines in Sichuan Province. Although the Chinese government has taken a series of policies and measures, such as designated production of medicines, monitoring of medicine shortages, and the establishment of centralized production bases to improve the availability of medicines (41). The lack of financial incentives to produce low-price anticancer medicines and weak and inefficient drug procurement and distribution systems might have an negative impact on the availability of children's essential anticancer medicines (42, 43).

Price was also an important factor hindering access to essential anticancer medicines. Similar to the other studies' results (18, 19), our study results showed that the MPR of the essential anticancer original brands for children was higher than generics. The prices of 87.5% (14/16) original brands and 41.9% (13/31) lowest-priced generics exceeded their 1.5 times IRP. National Basic Medical Insurance greatly improved the affordability of essential anticancer medicines for children in Sichuan Province. After medical insurance reimbursement, 78.9% (30/38) of lowest-priced generics and 50.0% (8/16) of original brands were considered affordable (the 30-day medicine expenses ≤10% of the monthly household income), and the number of affordable generics increased by 6 (25.0%) and original brands increased by 1 (14.3%). But there were still some medicines whose costs far exceeded the affordability of the patient's family after medical insurance reimbursement (e.g., rituximab injection, the 30-day medicine expenses even exceeded the average monthly household income).

Since 2018, the National Healthcare Security Administration, the National Health Commission, and the Ministry of Human Resources and Social Security have organized national negotiations for anticancer medicines to be incorporated into the medical insurance medicine catalog in China (44). According to reports, the average price of 14 anticancer medicines included in the 2020 medical insurance catalog dropped by 14.95%, of which some first-line anticancer medicines dropped by more than 60%, saving about 3 billion yuan for patients with cancer (45). The “Notice on the Applicable Diseases and Drug Recognition Standards for Single-path Payment Drugs and High-value Drugs in Sichuan Province in 2020” (46) announced that Pegaspargase injection as the first-line treatment medicine for children with acute lymphoblastic leukemia was included in the single-path payment of medical insurance, 70% reduction in patient out-of-pocket. Besides medical insurance, to reduce the price of anticancer medicines, China launched a national centralized drug procurement pilot in 2018, which required all regions to carry out special procurement bidding for anticancer medicines in the medical insurance catalog (47).

There were several limitations in this study. First, the medicine availability analysis was based on the 2020 medicine inventory data of public hospitals in Sichuan Province. As long as one medicine was stocked in a public sector for more than 1 day in the year, it was considered that the medicine was available in this public sector. The frequency and duration of medicines shortages were unclear, so this study might overestimate the availability of medicines. Second, only the availability of essential anticancer medicines for children was evaluated in tertiary public hospitals, not in the secondary and primary public hospitals, which might overestimate the availability of medicines. Because some studies and news had reported that anticancer medicines in secondary public hospitals were easier to shortages than in tertiary public hospitals and the availability was lower (39). Third, the medicine affordability was calculated based on the 30-day medicine expenses needed to treat a child with a body surface area of 1 m^2^ and a weight of 30 kg. There might be fluctuations in medicine affordability for children with other weights and body surface areas. In addition, cancer treatment usually used a combination of anticancer medicines for a long time. This study only calculated the affordability of a single medicine within 30 days, which might overestimate the affordability. Because most anticancer regimens would use a combination of multiple anticancer medicines, there is a situation that a single medicine that could be affordable but a combination medicine regimen could not. It was recommended that other researchers calculate the burden of childhood cancer diseases based on the medicare insurance database in future.

## Conclusion

Despite the availability of LPGs being higher than OBs in public hospitals in Sichuan Province, the availability of essential anticancer medicines for children was still low, which was similar to previous studies in low- and middle-income countries. In total of 45.2% lowest-priced generics and 87.5% of original brands exceeded their 1.5 times IRP. After medical insurance reimbursement, more than half of lowest-priced generics and original brands were affordable, but original brands had higher prices and lower affordability than generics. With the recent launch of various government health insurance policies, such as single-path payment, we recommend higher subsidies for essential medicines for cancer treatment to limit catastrophic health expenditures, such as biological medicine.

## Data availability statement

The original contributions presented in the study are included in the article/Supplementary material, further inquiries can be directed to the corresponding author.

## Author contributions

ZC and SL substantially contributed to the conception and design of the study, performed the analyses, and wrote the manuscript. All authors revised it critically for important intellectual content and gave their approval of the final version.

## Funding

This study was supported by Sichuan Province Science and Technology Plan Project (No. 2020YFS0035).

## Conflict of interest

The authors declare that the research was conducted in the absence of any commercial or financial relationships that could be construed as a potential conflict of interest.

The reviewer JP declared a shared affiliation with the authors to the handling editor at the time of review.

## Publisher's note

All claims expressed in this article are solely those of the authors and do not necessarily represent those of their affiliated organizations, or those of the publisher, the editors and the reviewers. Any product that may be evaluated in this article, or claim that may be made by its manufacturer, is not guaranteed or endorsed by the publisher.
