# Supplementary material for: Accessibility of essential anticancer medicines for children in the Sichuan Province of China
Source: Front Public Health. 2022 Nov 4;10:980969. doi: 10.3389/fpubh.2022.980969 (PMC9672812; doi:10.3389/fpubh.2022.980969)
Supplement: Supplementary file 1 [file Data_Sheet_1.docx]

**Supplementary Tables**

**Table 1 Distribution of surveyed public hospitals and the GDP per capita level of each city in Sichuan Province**

| **GDP Per Capita （USD）** | **City** | **No. General Hospital** | **No. Children’s Hospital** | **No. Cancer Hospital** |
| --- | --- | --- | --- | --- |
| **≥12535** | Chengdu | 30 | 15 | 1 |
|  | Panzhihua | 5 | 1 | 0 |
| **4046~12535** | Deyang | 5 | 1 | 0 |
|  | Mianyang | 10 | 1 | 0 |
|  | Yibin | 8 | 1 | 0 |
|  | Leshan | 3 | 1 | 0 |
|  | Zigong | 4 | 2 | 0 |
|  | Luzhou | 5 | 1 | 0 |
|  | Yaan | 3 | 0 | 0 |
|  | Meishan | 1 | 1 | 0 |
|  | Suining | 3 | 0 | 0 |
|  | Aba | 2 | 0 | 0 |
|  | Neijiang | 5 | 2 | 0 |
|  | Guangan | 3 | 0 | 0 |
|  | Nanchong | 9 | 0 | 0 |
|  | Dazhou | 5 | 1 | 0 |
|  | Guangyuan | 5 | 1 | 0 |
|  | Liangshan | 4 | 1 | 0 |
|  | Ganzi | 1 | 0 | 0 |
|  | Ziyang | 4 | 0 | 0 |
| **4045＜** | Bazhong | 5 | 2 | 0 |
| **Total Number** | | **120** | **31** | **1** |

**Table 2 List of surveyed essential medicines for treating childhood cancers**

| **Generic Name** | **Dosage Form** | **Strength** | **Whether in 2021 WHO EMLc** | **Whether in 2018 NEML** | **Medical Insurance Reimbursement Level** |
| --- | --- | --- | --- | --- | --- |
| Doxorubicin | Powder for injection | 10 mg | Y | Y | Class A |
| Doxorubicin | Powder for injection | 50 mg | Y | N | Class A |
| Cytarabine | Powder for injection | 100 mg | Y | Y | Class A |
| Cytarabine | Powder for injection | 50 mg | N | Y | Class A |
| Oxaliplatin | Powder for injection | 50 mg | Y | Y | Class B |
| Oxaliplatin | Powder for injection | 100 mg | Y | Y | Class B |
| Oxaliplatin | Injection | 50 mg/10 ml, 10 ml | Y | N | Class B |
| Oxaliplatin | Injection | 100 mg/20 ml, 20 ml | Y | N | Class B |
| Busulfan | Tablet | 0.5 mg | N | Y | Class A |
| Busulfan | Tablet | 2 mg | N | Y | Class A |
| Allopurinol | Tablet | 100 mg | Y | Y | Class A |
| Bleomycin | Powder for injection | 15 mg | Y | N | Class B |
| Dacarbazine | Powder for injection | 100 mg | Y | N | Class B |
| Dasatinib | Tablet | 20 mg | Y | N | Conditional Class B |
| Dasatinib | Tablet | 50 mg | Y | N | Conditional Class B |
| Dasatinib | Tablet | 70 mg | Y | N | Conditional Class B |
| Dasatinib | Tablet | 100 mg | Y | N | Conditional Class B |
| Dexamethasone | Tablet | 0.75 mg | N | Y | Class A |
| Dexamethasone | Injection | 2 mg/ml | N | Y | Class A |
| Dexamethasone | Injection | 5 mg/ml | N | Y | Class A |
| Fluorouracil | Injection | 50 mg/ml, 5 ml | Y | N | Class A |
| Fluorouracil | Injection | 0.25 g/10ml | N | Y | Class A |
| Cyclophosphamide | Powder for injection | 500 mg | Y | Y | Class A |
| Cyclophosphamide | Tablet | 50 mg | Y | Y | Class A |
| Cyclophosphamide | Powder for injection | 1 g | Y | N | Class A |
| Cyclophosphamide | Powder for injection | 100 mg | N | Y | Class A |
| Cyclophosphamide | Powder for injection | 200 mg | N | Y | Class A |
| Methotrexate | Tablet | 2.5 mg | Y | Y | Class A |
| Methotrexate | Powder for injection | 50 mg | Y | N | Class A |
| Methotrexate | Powder for injection | 5 mg | N | Y | Class A |
| Methotrexate | Powder for injection | 100 mg | N | Y | Class A |
| Methylprednisolone | Tablet | 4 mg | N | Y | Class A |
| Methylprednisolone | Powder for injection | 40 mg | N | Y | Class B |
| Methylprednisolone | Powder for injection | 500 mg | N | Y | Class B |
| Carboplatin | Injection | 150 mg/15 ml | Y | N | Class A |
| Carboplatin | Powder for injection | 50 mg | N | Y | Class A |
| Carboplatin | Powder for injection | 100 mg | N | Y | Class A |
| Rasburicase | Powder for injection | 1.5 mg | Y | N | N |
| Rituximab | Injection | 100 mg/10 ml | Y | Y | Conditional Class B |
| Rituximab | Injection | 500 mg/50 ml | Y | Y | Conditional Class B |
| Mesna | Injection | 100 mg/ml, 4 ml | Y | Y | Class B |
| Mesna | Injection | 100 mg/ml, 10 ml | Y | N | Class B |
| Asparaginase | Powder for injection | 10000 IU | Y | Y | Class A |
| Asparaginase | Powder for injection | 5000 IU | N | Y | Class A |
| Nilotinib | Capsule | 150 mg | Y | N | N |
| Nilotinib | Capsule | 200 mg | Y | N | N |
| Pegaspargase | Injection | 3750 IU, 5ml | Y | Y | Conditional Class A |
| Pegaspargase | Injection | 1500 IU,2ml | N | Y | Conditional Class A |
| Bleomycin A5 | Powder for injection | 4 mg | N | Y | Class A |
| Bleomycin A5 | Powder for injection | 8 mg | N | Y | Class A |
| Prednisolone | Tablet | 5 mg | Y | N | Class B |
| Hydroxycarbamide | Tablet | 500 mg | Y | Y | N |
| Hydroxycarbamide | Tablet | 200 mg | Y | N | N |
| Hydroxycarbamide | Tablet | 250 mg | Y | N | N |
| Hydrocortisone | Powder for injection | 100 mg | Y | Y | Class A |
| Hydrocortisone | Tablet | 10 mg | N | Y | Class A |
| Hydrocortisone | Tablet | 20 mg | N | Y | Class A |
| Hydrocortisone | Injection | 10 mg/2 ml | N | Y | Class A |
| Hydrocortisone | Injection | 25 mg/5 ml | N | Y | Class A |
| Hydrocortisone | Injection | 100 mg/20 ml | N | Y | Class A |
| Hydrocortisone | Powder for injection | 50 mg | N | Y | Class A |
| Mercaptopurine | Tablet | 50 mg | Y | Y | Class A |
| Mercaptopurine | Tablet | 25 mg | N | Y | Class A |
| All-trans retinoid acid (ATRA) | Capsule | 10 mg | Y | Y | Class A |
| Daunorubicin | Powder for injection | 20 mg | N | Y | Class A |
| Arsenic trioxide | Injection | 1 mg/ml | Y | Y | Class B |
| Arsenic trioxide | Powder for injection | 5 mg | N | Y | Class B |
| Arsenic trioxide | Powder for injection | 10 mg | N | Y | Class B |
| Calcium folinate | Injection | 3 mg/ml,10 ml | Y | N | Class A |
| Calcium folinate | Tablet | 5 mg | Y | N | Class A |
| Calcium folinate | Tablet | 15 mg | Y | N | Class A |
| Calcium folinate | Tablet | 25 mg | Y | N | Class A |
| Calcium folinate | Injection | 100 mg/10 ml | N | Y | Class A |
| Calcium folinate | Powder for injection | 25 mg | N | Y | Class A |
| Calcium folinate | Powder for injection | 50 mg | N | Y | Class A |
| Calcium folinate | Powder for injection | 100 mg | N | Y | Class A |
| Irinotecan | Injection | 40 mg/2 ml | Y | N | Class B |
| Irinotecan | Injection | 100 mg/5 ml | Y | N | Class B |
| Imatinib | Tablet | 100 mg | Y | Y | Conditional Class B |
| Imatinib | Tablet | 400 mg | Y | Y | Conditional Class B |
| Imatinib | Capsule | 0.05 g | N | Y | Conditional Class B |
| Etoposide | Injection | 20 mg/ml, 5 ml | Y | Y | Class A |
| Etoposide | Capsule | 50 mg | Y | N | Class B |
| Etoposide | Injection | 40 mg/2 ml | N | Y | Class A |
| Everolimus | Tablet | 2.5 mg | Y | N | Conditional Class B |
| Everolimus | Tablet | 5 mg | Y | N | Conditional Class B |
| Everolimus | Tablet | 10 mg | Y | N | Conditional Class B |
| Ifosfamide | Powder for injection | 500 mg | Y | Y | Class B |
| Ifosfamide | Powder for injection | 1 g | Y | Y | Class B |
| Vinorelbine | Capsule | 20 mg | Y | N | Class B |
| Vinorelbine | Injection | 10 mg/ml, 1 ml | Y | N | Class B |
| Vinorelbine | Injection | 50 mg/5 ml | Y | N | Class B |
| Vincristine | Injection | 1 mg/ml | Y | Y | Class A |
| Vincristine | Powder for injection | 1 mg | Y | N | Class A |
| Paclitaxel | Injection | 6 mg/ml | Y | Y | Class A |

**Y:** Yes; **N:** No

**Table 3 Recommended medicines, doses and duration for different cancers**

| **Disease** | **Medicine** | **Dosage and Duration** |
| --- | --- | --- |
| Acute lymphoblastic leukaemia ^1^ | Asparaginase^2^ | 25000 IU/m^2^, 3 times a week (d1/d3/d5), 6 times in total |
|  | Cyclophosphamide^3^ | Injection: 10-15 mg/kg, once a week, 2 times in a row, repeat after 1-2 weeks  Oral: 2-6 mg/kg/d for 10-14 days, repeat after 1-2 weeks |
|  | Cytarabine^4^ | 200 mg/m^2^/d, 5 days, once per 4 weeks |
|  | Daunorubicin^5^ | 45 mg/m^2^/d, Days 1-3 |
|  | Dexamethasone^6^ | 0.02-0.3 mg/kg/d or 0.6-9 mg/m^2^/d |
|  | Doxorubicin^7^ | 60-75 mg/m^2^, repeated per 3-4 weeks |
|  | Etoposide^8^ | 60-100 mg/m^2^/d, 5 consecutive days, once per 4 weeks |
|  | Hydrocortisone^9^ | 20-25 mg/m^2^/d |
|  | Imatinib^10^ | 600 mg/d |
|  | Mercaptopurine^11^ | 2.5 mg/kg/d or 80-100 mg/m^2^/d |
|  | Methotrexate^12^ | Oral/Injection: 30 mg/m^2^, once a week |
|  | Methylprednisolone^13^ | 4-48 mg/d |
|  | Pegaspargase^14^ | 2500 IU/m^2^/14d |
|  | Vincristine^15^ | 0.05-0.075 mg/kg, once a week for 3-4 weeks |
| Non-Hodgkin lymphoma^16^ | Cyclophosphamide^3^ | Injection: 10-15 mg/kg, once a week, 2 times in a row, repeat after 1-2 weeks  Oral: 2-6 mg/kg/d for 10-14 days, repeat after 1-2 weeks |
|  | Cytarabine^17^ | 100 mg/m^2^/d as a continuous infusion over 24 hours on Days 2 through 6 (5 days; total dose: 500 mg/m^2^ as a 120-hour infusion), twice per four weeks |
|  | Dexamethasone^6^ | 0.02-0.3 mg/kg/d or 0.6-9 mg/m^2^/d |
|  | Doxorubicin^7^ | 60-75 mg/m^2^, repeated per 3-4 weeks |
|  | Etoposide^8^ | 100 mg/m/d, Days 1-3 |
|  | Hydrocortisone^9^ | 20-25 mg/m^2^/d |
|  | Ifosfamide^18^ | 1.2-2.4g/m^2^/d, the maximum dose is 60mg/kg, continuous use for 5 days |
|  | Mesna^19^ | 1800 mg/m^2^/day to 5000 mg/m^2^/day as a continuous infusion (100% of the ifosfamide dose), repeated each day ifosfamide is received |
|  | Methotrexate^12^ | Oral/Injection: 30 mg/m^2^, once a week |
|  | Methylprednisolone^13^ | 4-48 mg/d |
|  | Prednisolone^20^ | Oral: 5-60 mg/d (usually 40 mg/m^2^/d), 3-4 weeks |
|  | Vincristine^15^ | 0.05-0.075 mg/kg, once a week for 3-4 weeks |
|  | Rituximab^21^ | 375 mg/m^2^，once per 2 weeks |
| Hodgkin lymphoma^22^ | Bleomycin^23^ | 10-20 IU/m^2^ or 0.25-0.5 IU/kg., once a week |
|  | Cyclophosphamide^3^ | Injection: 10-15 mg/kg, once a week, 2 times in a row, repeat after 1-2 weeks  Oral: 2-6 mg/kg/d for 10-14 days, repeat after 1-2 weeks |
|  | Ifosfamide^18^ | 1.2-2.4g/m^2^/d, the maximum dose is 60mg/kg, continuous use for 5 days |
|  | Mesna^19^ | 1800 mg/m^2^/day to 5000 mg/m^2^/day as a continuous infusion (100% of the ifosfamide dose), repeated each day ifosfamide is received |
|  | Dacarbazine^16^ | 250 mg/m^2^/d, Days 1-5, once per 4 weeks |
|  | Doxorubicin^7^ | 60-75 mg/m^2^, repeated per 3-4 weeks |
|  | Etoposide^8^ | Injection: 60-100mg/m^2^/d, 5 consecutive days, once per 4 weeks |
|  | Prednisolone^20^ | Oral: 5-60 mg/d (usually 40 mg/m^2^/d), 3-4 weeks |
|  | Vincristine^15^ | 0.05-0.075 mg/kg, once a week for 3-4 weeks |
| Medulloblastoma ^24-25^ | Carboplatin^26^ | 560mg/m^2^, Day 1, once per 4 weeks |
|  | Vincristine^15^ | 0.05-0.075 mg/kg, once a week for 3-4 weeks |
|  | Etoposide^8^ | 60-100 mg/m^2^/d, 3-5 consecutive days, repeat after 3-4 weeks |
|  | Methotrexate^12^ | Oral/Injection: 30 mg/m^2^, once a week |
|  | Cyclophosphamide^3^ | Injection: 10-15 mg/kg, once a week, 2 times in a row, repeat after 1-2 weeks  Oral: 2-6 mg/kg/d for 10-14 days, repeat after 1-2 weeks |
| Nephroblastoma (Wilms tumour) ^24^ | Carboplatin^26^ | 400mg/m^2^, Day 1, once per 21 days |
|  | Vincristine^15^ | 0.05-0.075 mg/kg, once a week for 3-4 weeks |
|  | Dactinomycin^27^ | 0.45 mg/m^2^/d，5d/w，3-6 week |
|  | Doxorubicin^7^ | 60-75 mg/m^2^, repeated per 3-4 weeks |
|  | Etoposide^8^ | 100 mg/m^2^/day, Day 1, 3, and 5, repeated 3-4 weeks later |
|  | Cyclophosphamide^3^ | Injection: 10-15 mg/kg, once a week, 2 times in a row, repeat after 1-2 weeks  Oral: 2-6 mg/kg/d for 10-14 days, repeat after 1-2 weeks |
|  | Ifosfamide^18^ | 1.2-2.4g/m^2^/d, the maximum dose is 60mg/kg, continuous use for 5 days |
|  | Irinotecan^27^ | 80mg/m^2^/d，Day 1, once per 1-3 week |
|  | Mesna^19^ | 1800 mg/m^2^/day to 5000 mg/m^2^/day as a continuous infusion (100% of the ifosfamide dose), repeated each day ifosfamide is received |

**References**

1. Up To Date: Overview of the treatment of acute lymphoblastic leukemia/lymphoma in children and adolescents. In. Available: https://cams.du2022.top/contents/overview-of-the-treatment-of-acute-lymphoblastic-leukemia-lymphoma-in-children-and-adolescents. [Accessed 13 January 2022]

2. Up To Date: Asparaginase. In. Available: https://cams.du2022.top/contents/zh-Hans/92806?search=%E6%80%A5%E6%80%A7%E6%B7%8B%E5%B7%B4%E7%BB%86%E8%83%9E%E7%99%BD%E8%A1%80%E7%97%85&topicRef=6245&source=see_link. [Accessed 13 January 2022]

3. Up To Date: Cyclophosphamide. In. Available: https://cams.du2022.top/contents/zh-Hans/92845?kp_tab=drug_dxy&display_rank=1&search=cyclophosphamide&selectedTitle=1~149&source=panel_search_result. [Accessed 13 January 2022]

4. Up To Date: Cytarabine. In. Available: https://cams.du2022.top/contents/zh-Hans/92802?kp_tab=drug_dxy&display_rank=1&search=cytarabine&selectedTitle=1~149&source=panel_search_result. [Accessed 13 January 2022]

5. Up To Date: Daunorubicin. In. Available: https://cams.du2022.top/contents/zh-Hans/92777?kp_tab=drug_dxy&display_rank=1&search=daunorubicin&selectedTitle=1~115&source=panel_search_result. [Accessed 13 January 2022]

6. Up To Date: Dexamethasone. In. Available: https://cams.du2022.top/contents/zh-Hans/92098?kp_tab=drug_dxy&display_rank=1&search=dexamethasone&selectedTitle=1~146&source=panel_search_result. [Accessed 13 January 2022]

7. Up To Date: Doxorubicin. In. Available: https://cams.du2022.top/contents/zh-Hans/92774?kp_tab=drug_dxy&display_rank=1&search=doxorubicin&selectedTitle=1~147&source=panel_search_result. [Accessed 13 January 2022]

8. Up To Date: Etoposide. In. Available: https://cams.du2022.top/contents/zh-Hans/92839?kp_tab=drug_dxy&display_rank=1&search=etoposide&selectedTitle=1~149&source=panel_search_result. [Accessed 13 January 2022]

9. Up To Date: Hydrocortisone. In. Available: https://cams.du2022.top/contents/zh-Hans/92101?kp_tab=drug_dxy&display_rank=1&search=hydrocortisone&selectedTitle=1~146&source=panel_search_result. [Accessed 13 January 2022]

10. Up To Date: Imatinib. In. Available: https://cams.du2022.top/contents/zh-Hans/125749?kp_tab=drug_dxy&display_rank=1&search=Imatinib&selectedTitle=1~131&source=panel_search_result. [Accessed 13 January 2022]

11. Up To Date: Mercaptopurine. In. Available: https://cams.du2022.top/contents/zh-Hans/92793?kp_tab=drug_dxy&display_rank=1&search=mercaptopurine&selectedTitle=1~131&source=panel_search_result. [Accessed 13 January 2022]

12. Up To Date: Methotrexate. In. Available: https://cams.du2022.top/contents/zh-Hans/92799?kp_tab=drug_dxy&display_rank=1&search=methotrexate&selectedTitle=1~149&source=panel_search_result. [Accessed 13 January 2022]

13. Up To Date: Methylprednisolone. In. Available: https://cams.du2022.top/contents/zh-Hans/92105?kp_tab=drug_dxy&display_rank=1&search=methylprednisolone&selectedTitle=1~149&source=panel_search_result. [Accessed 13 January 2022]

14. Up To Date: Pegaspargase. In. Available: https://cams.du2022.top/contents/zh-Hans/92777?kp_tab=drug_dxy&display_rank=1&search=daunorubicin&selectedTitle=1~115&source=panel_search_result. [Accessed 13 January 2022]

15. Up To Date: Vincristine. In. Available: https://cams.du2022.top/contents/zh-Hans/126725?kp_tab=drug_dxy&display_rank=1&search=vincristine&selectedTitle=1~147&source=panel_search_result. [Accessed 13 January 2022]

16. Up To Date: Dacarbazine. In. Available: <https://cams.du2022.top/contents/zh-Hans/92851?search=%E9%9C%8D%E5%A5%87%E9%87%91%E6%B7%8B%E5%B7%B4%E7%98%A4&topicRef=6246&source=see_link>. [Accessed 14 January 2022]

17. Up To Date: Cytarabine (conventional): Pediatric drug information. In. Available: https://cams.du2022.top/contents/cytarabine-conventional-pediatric-drug-information?search=%E9%98%BF%E7%B3%96%E5%AD%A2%E8%8B%B7&source=panel_search_result&selectedTitle=2~148&usage_type=panel&kp_tab=drug_foreign&display_rank=1. [Accessed 13 January 2022]

18. Up To Date: Ifosfamide. In. Available: https://cams.du2022.top/contents/zh-Hans/92842?topicRef=13940&source=see_link. [Accessed 13 January 2022]

19. Up To Date: Mesna: Pediatric drug information. In. Available: https://cams.du2022.top/contents/mesna-pediatric-drug-information?search=mesna&source=panel_search_result&selectedTitle=3~70&usage_type=panel&kp_tab=drug_foreign&display_rank=1. [Accessed 14 January 2022]

20. Up To Date: Prednisolone. In. Available: https://cams.du2022.top/contents/zh-Hans/92104?kp_tab=drug_dxy&display_rank=1&search=prednisolone&selectedTitle=1~146&source=panel_search_result. [Accessed 13 January 2022]

21. Up To Date: Rituximab. In. Available: https://cams.du2022.top/contents/zh-Hans/92827?search=%E9%9D%9E%E9%9C%8D%E5%A5%87%E9%87%91%E6%B7%8B%E5%B7%B4%E7%98%A4&topicRef=13940&source=see_link. [Accessed 14 January 2022]

22. Up To Date: Overview of Hodgkin lymphoma in children and adolescents. In. Available: https://cams.du2022.top/contents/overview-of-hodgkin-lymphoma-in-children-and-adolescents. [Accessed 13 January 2022]

23. Up To Date: Bleomycin. In. Available: https://cams.du2022.top/contents/zh-Hans/94067?search=%E9%9C%8D%E5%A5%87%E9%87%91%E6%B7%8B%E5%B7%B4%E7%98%A4&topicRef=6246&source=see_link. [Accessed 14 January 2022]

24. Guidelines for the diagnosis and treatment of medulloblastoma in children (2021). *Clinical Education of General Practice*  2021;19(07):581-84. doi: 10.13558/j.cnki.issn1672-3686.2021.007.002.

25. MD PhD Scott L Pomeroy. Up To Date: Treatment and prognosis of medulloblastoma. In, 2022. Available: https://www-uptodate-com-s--cams1.du2022.top/contents/treatment-and-prognosis-of-medulloblastoma. [Accessed 13 January 2022]

26. Up To Date: Carboplatin. In. Available: https://cams.du2022.top/contents/zh-Hans/94068?kp_tab=drug_dxy&display_rank=1&search=%E5%8D%A1%E9%93%82&selectedTitle=1~149&source=panel_search_result. [Accessed 14 January 2022]

27. Up To Date: Dactinomycin. In. Available: https://cams.du2022.top/contents/zh-Hans/92776?search=%E8%82%BE%E6%AF%8D%E7%BB%86%E8%83%9E%E7%98%A4%E5%84%BF%E7%AB%A5&topicRef=6237&source=see_link. [Accessed 14 January 2022]

**Table 4 Availability of essential anticancer medicines for children in public hospitals in Sichuan Province**

| **Medicine Generic Name** | **Dosage Form** | **Strength** | **Availability of Generics** | | **Availability of Original Brands** | |
| --- | --- | --- | --- | --- | --- | --- |
|  |  |  | **Number of Public Hospitals (n)** | **Percentage of Public Hospitals (%)** | **Number of Public Hospitals (n)** | **Percentage of Public Hospitals (%)** |
| Doxorubicin | Powder for injection | 10 mg | 60 | 39.5% | 5 | 3.3% |
| Doxorubicin | Powder for injection | 50 mg | 0 | 0.0% | 0 | 0.0% |
| Cytarabine | Powder for injection | 100 mg | 56 | 36.8% | 15 | 9.9% |
| Cytarabine | Powder for injection | 50 mg | 2 | 1.3% | 0 | 0.0% |
| Oxaliplatin | Powder for injection | 50 mg | 108 | 71.1% | 32 | 21.1% |
| Oxaliplatin | Powder for injection | 100 mg | 24 | 15.8% | 0 | 0.0% |
| Oxaliplatin | Injection | 50 mg/10 ml, 10 ml | 0 | 0.0% | 0 | 0.0% |
| Oxaliplatin | Injection | 100 mg/20 ml, 20 ml | 0 | 0.0% | 0 | 0.0% |
| Busulfan | Tablet | 0.5 mg | 0 | 0.0% | 0 | 0.0% |
| Busulfan | Tablet | 2 mg | 0 | 0.0% | 0 | 0.0% |
| Allopurinol | Tablet | 100 mg | 51 | 33.6% | 0 | 0.0% |
| Bleomycin | Powder for injection | 15 mg | 49 | 32.2% | 2 | 1.3% |
| Dacarbazine | Powder for injection | 100 mg | 52 | 34.2% | 0 | 0.0% |
| Dasatinib | Tablet | 20 mg | 10 | 6.6% | 0 | 0.0% |
| Dasatinib | Tablet | 50 mg | 34 | 22.4% | 1 | 0.7% |
| Dasatinib | Tablet | 70 mg | 0 | 0.0% | 0 | 0.0% |
| Dasatinib | Tablet | 100 mg | 0 | 0.0% | 0 | 0.0% |
| Dexamethasone | Tablet | 0.75 mg | 139 | 91.4% | 0 | 0.0% |
| Dexamethasone | Injection | 2 mg/ml | 9 | 5.9% | 0 | 0.0% |
| Dexamethasone | Injection | 5 mg/ml | 147 | 96.7% | 0 | 0.0% |
| Fluorouracil | Injection | 50 mg/ml, 5 ml | 0 | 0.0% | 0 | 0.0% |
| Fluorouracil | Injection | 0.25 g/10ml | 99 | 65.1% | 0 | 0.0% |
| Cyclophosphamide | Powder for injection | 500 mg | 0 | 0.0% | 0 | 0.0% |
| Cyclophosphamide | Tablet | 50 mg | 0 | 0.0% | 0 | 0.0% |
| Cyclophosphamide | Powder for injection | 1 g | 0 | 0.0% | 0 | 0.0% |
| Cyclophosphamide | Powder for injection | 100 mg | 0 | 0.0% | 0 | 0.0% |
| Cyclophosphamide | Powder for injection | 200 mg | 89 | 58.6% | 49 | 32.2% |
| Methotrexate | Tablet | 2.5 mg | 101 | 66.4% | 0 | 0.0% |
| Methotrexate | Powder for injection | 50 mg | 1 | 0.7% | 0 | 0.0% |
| Methotrexate | Powder for injection | 5 mg | 57 | 37.5% | 0 | 0.0% |
| Methotrexate | Powder for injection | 100 mg | 59 | 38.8% | 0 | 0.0% |
| Methylprednisolone | Tablet | 4 mg | 31 | 20.4% | 64 | 42.1% |
| Methylprednisolone | Powder for injection | 40 mg | 117 | 77.0% | 53 | 34.9% |
| Methylprednisolone | Powder for injection | 500 mg | 6 | 3.9% | 59 | 38.8% |
| Carboplatin | Injection | 150 mg/15 ml | 0 | 0.0% | 4 | 2.6% |
| Carboplatin | Powder for injection | 50 mg | 13 | 8.6% | 0 | 0.0% |
| Carboplatin | Powder for injection | 100 mg | 14 | 9.2% | 0 | 0.0% |
| Rasburicase | Powder for injection | 1.5 mg | 0 | 0.0% | 0 | 0.0% |
| Rituximab | Injection | 100 mg/10 ml | 0 | 0.0% | 33 | 21.7% |
| Rituximab | Injection | 500 mg/50 ml | 0 | 0.0% | 25 | 16.4% |
| Mesna | Injection | 100 mg/ml, 4 ml | 74 | 48.7% | 0 | 0.0% |
| Mesna | Injection | 100 mg/ml, 10 ml | 0 | 0.0% | 0 | 0.0% |
| Asparaginase | Powder for injection | 10000 IU | 18 | 11.8% | 0 | 0.0% |
| Asparaginase | Powder for injection | 5000 IU | 5 | 3.3% | 0 | 0.0% |
| Nilotinib | Capsule | 150 mg | 0 | 0.0% | 0 | 0.0% |
| Nilotinib | Capsule | 200 mg | 0 | 0.0% | 0 | 0.0% |
| Pegaspargase | Injection | 3750 IU, 5ml | 19 | 12.5% | 0 | 0.0% |
| Pegaspargase | Injection | 1500 IU, 2ml | 0 | 0.0% | 0 | 0.0% |
| Bleomycin A5 | Powder for injection | 4 mg | 0 | 0.0% | 0 | 0.0% |
| Bleomycin A5 | Powder for injection | 8 mg | 38 | 25.0% | 0 | 0.0% |
| Prednisolone | Tablet | 5 mg | 0 | 0.0% | 0 | 0.0% |
| Hydroxycarbamide | Tablet | 500 mg | 46 | 30.3% | 0 | 0.0% |
| Hydroxycarbamide | Tablet | 200 mg | 0 | 0.0% | 0 | 0.0% |
| Hydroxycarbamide | Tablet | 250 mg | 0 | 0.0% | 0 | 0.0% |
| Hydrocortisone | Powder for injection | 100 mg | 16 | 10.5% | 0 | 0.0% |
| Hydrocortisone | Tablet | 10 mg | 0 | 0.0% | 0 | 0.0% |
| Hydrocortisone | Tablet | 20 mg | 6 | 3.9% | 0 | 0.0% |
| Hydrocortisone | Injection | 10 mg/2 ml | 34 | 22.4% | 0 | 0.0% |
| Hydrocortisone | Injection | 25 mg/5 ml | 1 | 0.7% | 0 | 0.0% |
| Hydrocortisone | Injection | 100 mg/20 ml | 36 | 23.7% | 0 | 0.0% |
| Hydrocortisone | Powder for injection | 50 mg | 122 | 80.3% | 0 | 0.0% |
| Mercaptopurine | Tablet | 50 mg | 6 | 3.9% | 0 | 0.0% |
| Mercaptopurine | Tablet | 25 mg | 0 | 0.0% | 0 | 0.0% |
| All-trans retinoid acid (ATRA) | Capsule | 10 mg | 50 | 32.9% | 0 | 0.0% |
| Daunorubicin | Powder for injection | 20 mg | 52 | 34.2% | 5 | 3.3% |
| Arsenic trioxide | Injection | 1 mg/ml | 32 | 21.1% | 0 | 0.0% |
| Arsenic trioxide | Powder for injection | 5 mg | 0 | 0.0% | 0 | 0.0% |
| Arsenic trioxide | Powder for injection | 10 mg | 17 | 11.2% | 0 | 0.0% |
| Calcium folinate | Injection | 3 mg/ml,10 ml | 0 | 0.0% | 0 | 0.0% |
| Calcium folinate | Tablet | 5 mg | 0 | 0.0% | 0 | 0.0% |
| Calcium folinate | Tablet | 15 mg | 2 | 1.3% | 0 | 0.0% |
| Calcium folinate | Tablet | 25 mg | 0 | 0.0% | 0 | 0.0% |
| Calcium folinate | Injection | 100 mg/10 ml | 25 | 16.4% | 0 | 0.0% |
| Calcium folinate | Powder for injection | 25 mg | 2 | 1.3% | 0 | 0.0% |
| Calcium folinate | Powder for injection | 50 mg | 11 | 7.2% | 0 | 0.0% |
| Calcium folinate | Powder for injection | 100 mg | 104 | 68.4% | 0 | 0.0% |
| Irinotecan | Injection | 40 mg/2 ml | 33 | 21.7% | 2 | 1.3% |
| Irinotecan | Injection | 100 mg/5 ml | 21 | 13.8% | 4 | 2.6% |
| Imatinib | Tablet | 100 mg | 76 | 50.0% | 15 | 9.9% |
| Imatinib | Tablet | 400 mg | 0 | 0.0% | 0 | 0.0% |
| Imatinib | Capsule | 0.05 g | 0 | 0.0% | 0 | 0.0% |
| Etoposide | Injection | 20 mg /ml, 5 ml | 108 | 71.1% | 0 | 0.0% |
| Etoposide | Capsule | 50 mg | 32 | 21.1% | 0 | 0.0% |
| Etoposide | Injection | 40 mg/2 ml | 6 | 3.9% | 0 | 0.0% |
| Everolimus | Tablet | 2.5 mg | 0 | 0.0% | 0 | 0.0% |
| Everolimus | Tablet | 5 mg | 0 | 0.0% | 2 | 1.3% |
| Everolimus | Tablet | 10 mg | 0 | 0.0% | 0 | 0.0% |
| Ifosfamide | Powder for injection | 500 mg | 59 | 38.8% | 0 | 0.0% |
| Ifosfamide | Powder for injection | 1 g | 19 | 12.5% | 4 | 2.6% |
| Vinorelbine | Capsule | 20 mg | 17 | 11.2% | 0 | 0.0% |
| Vinorelbine | Injection | 10 mg/ml, 1 ml | 64 | 42.1% | 2 | 1.3% |
| Vinorelbine | Injection | 50 mg/5 ml | 0 | 0.0% | 0 | 0.0% |
| Vincristine | Injection | 1 mg/ml | 0 | 0.0% | 0 | 0.0% |
| Vincristine | Powder for injection | 1 mg | 63 | 41.4% | 0 | 0.0% |
| Paclitaxel | Injection | 6 mg/ml | 126 | 82.9% | 6 | 3.9% |

**Table 5 Average availability of children’s essential anticancer medicines in the public hospital in Sichuan Province: comparison among regions with different GDP per capita levels**

| **Medicine Type** | | **Average Availability (％)** | | | **Median Availability (％)** | | |
| --- | --- | --- | --- | --- | --- | --- | --- |
|  |  | **GDP Per Capita**  **≤4045 USD**  **（n =7）** | **4046 USD ＜GDP Per Capita**  **≤12535 USD**  **（n =93）** | **GDP Per Capita**  **＞12535 USD**  **（n=52）** | **GDP Per Capita**  **≤4045 USD**  **（n =7）** | **4046 USD ＜GDP Per Capita**  **≤12535 USD**  **（n =93）** | **GDP Per Capita**  **＞12535 USD**  **（n=52）** |
| **Originator Brand** | WHO EMLc  (n=59) | 0.7% | 1.6% | 2.0% | 0.0% | 0.0% | 0.0% |
|  | Non-WHO EMLc  (n=36) | 2.8% | 4.2% | 4.5% | 0.0% | 0.0% | 0.0% |
|  | NEM  (n=61) | 2.3% | 3.9% | 4.2% | 0.0% | 0.0% | 0.0% |
|  | Non-NEM  (n=34) | 0.0% | 0.2% | 0.6% | 0.0% | 0.0% | 0.0% |
|  | Class A Medical Insurance  (n=53) | 2.7% | 1.7% | 2.0% | 0.0% | 0.0% | 0.0% |
|  | Class B Medical Insurance  (n=36) | 0.0% | 4.3% | 4.8% | 0.0% | 0.0% | 0.0% |
|  | Non-Medical Insurance  (n=6) | 0.0% | 0.0% | 0.0% | 0.0% | 0.0% | 0.0% |
|  | Total  (n=95) | 1.5% | 2.6% | 2.9% | 0.0% | 0.0% | 0.0% |
| **Generic** | WHO EMLc  (n=59) | 11.9% | 16.3% | 15.7% | 0.0% | 0.0% | 1.9% |
|  | Non-WHO EMLc  (n=36) | 21.0% | 22.4% | 23.4% | 0.0% | 6.5% | 9.6% |
|  | NEM  (n=61) | 21.1% | 24.9% | 24.8% | 0.0% | 11.8% | 13.5% |
|  | Non-NEM  (n=34) | 5.0% | 7.4% | 7.5% | 0.0% | 0.0% | 0.0% |
|  | Class A Medical Insurance  (n=53) | 18.9% | 21.6% | 22.6% | 0.0% | 5.4% | 7.7% |
|  | Class B Medical Insurance  (n=36) | 11.9% | 16.2% | 15.5% | 0.0% | 8.1% | 5.8% |
|  | Non-Medical Insurance  (n=6) | 4.8% | 6.6% | 2.2% | 0.0% | 0.0% | 0.0% |
|  | Total  (n=95) | 15.3% | 18.6% | 18.6% | 0.0% | 5.4% | 3.8% |

**Table 6 Affordability of children’s essential anticancer medicines in public hospitals in Sichuan Province before and after medical insurance reimbursement**

|  | | **Number of Affordable Medicines Before Medical Insurance Reimbursement** | | | **Number of Affordable Medicines After Medical Insurance Reimbursement** | | | ***P*-Value*** |
| --- | --- | --- | --- | --- | --- | --- | --- | --- |
|  |  | **Affordability(≤10%)** | **Poor Affordability**  **(10%~25%)** | **Very Poor Affordability**  **(＞25%)** | **Affordability(≤10%)** | **Poor Affordability**  **(10%~25%)** | **Very Poor Affordability**  **(＞25%)** |  |
| Lowest-Priced Generics  (n=38) | Urban Residents | 26 | 4 | 8 | 30 | 7 | 1 | 0.170 |
|  | Rural Residents | 22 | 4 | 12 | 25 | 5 | 8 | 0.392 |
|  | All Residents | 24 | 6 | 8 | 30 | 6 | 2 | 0.085 |
| Original Brands  (n=16) | Urban Residents | 7 | 3 | 6 | 10 | 1 | 5 | 0.468 |
|  | Rural Residents | 3 | 4 | 9 | 7 | 3 | 6 | 0.210 |
|  | All Residents | 7 | 3 | 6 | 8 | 2 | 6 | 0.867 |

*Affordability comparison of medicines before and after medical insurance reimbursement.
